# Eating disorder features in indigenous Aboriginal and Torres Strait Islander Australian Peoples

**DOI:** 10.1186/1471-2458-12-233

**Published:** 2012-03-23

**Authors:** Phillipa J Hay, Chris Carriage

**Affiliations:** 1 School of Medicine, University of Western Sydney, Locked bag 1797, Penrith 2750, NSW, Australia; 2 School of Medicine, James Cook University, Townsville, Queensland, Australia

## Abstract

**Background:**

Obesity and related cardiovascular and metabolic conditions are well recognized problems for Australian Aboriginal and Torres Strait Islander peoples. However, there is a dearth of research on relevant eating disorders (EDs) such as binge eating disorder in these groups.

**Methods:**

Data were obtained from interviews of 3047 (in 2005) and 3034 (in 2008) adults who were participants in a randomly selected South Australian household survey of individuals' age > 15 years. The interviewed comprised a general health survey in which ED questions were embedded. Data were weighted according to national census results and comprised key features of ED symptoms.

**Results:**

In 2005 there were 94 (85 weighted) First Australian respondents, and in 2008 65 (70 weighted). Controlling for secular differences, in 2005 rates of objective binge eating and levels of weight and shape influence on self-evaluation were significantly higher in indigenous compared to non-indigenous participants, but no significant differences were found in ED features in 2008.

**Conclusions:**

Whilst results on small numbers must be interpreted with caution, the main finding was consistent over the two samples. For First Australians ED symptoms are at least as frequent as for non-indigenous Australians.

## Background

Aboriginal and Torres Strait Islander peoples are well known to have higher risk for a range of disorders related to poor nutrition, namely a diet high in refined carbohydrates and saturated fats. This leads to subsequent increased risk for obesity, Type 2 diabetes, cardiovascular disease and renal disease [1]. In addition there are higher rates of admissions for mental health problems and self-harm in indigenous Australians [1]. Research into mental health problems closely related to nutrition and obesity such as disordered eating and binge eating is minimal within Australia. However, international research indicates that these problems are prevalent in other indigenous cultures. A recent review of eating disorders in North American indigenous peoples found disordered eating and weight control behaviours, body dissatisfaction and eating disorders (where measured) were as frequent in First Nations or Inuit peoples as in White or other Americans [2]. One of the eight studies reviewed was a national US study of young adult women that in particular reported a higher frequency of indigenous women having problems with uncontrolled and/or 'embarrassing overeating' compared to non-indigenous [3]. The studies in this review were however confined to adolescent samples or College recruited samples [2]. Of concern, indigenous people were less likely to be referred on for further evaluation of their eating problems.

Further controlled [4] and uncontrolled [5,6] studies in Canada and the US have found high body dissatisfaction (desire to be thinner) and increased risk for an eating disorder in indigenous peoples, particularly youth. A recent large (10,334 participants, mean age 21.93 years) national US survey [7] of adult men and women has found higher rates of current binge eating, embarrassment about excessive eating, loss of control over eating, higher weight, but not a self-reported diagnosis of an eating disorder or use of inappropriate weight control behaviours in American Indian or Native American men (n = 236) and women (n = 253).

In Australia studies of indigenous peoples are confined to adolescent samples and have been focussed on body image disturbance. An Australian report found higher levels of body dissatisfaction and a desire to build up muscle in indigenous males but lower or similar levels of body dissatisfaction in female indigenous adolescents compared to non-indigenous. Both indigenous groups had greater engagement with weight control practices [8] but indigenous females appeared less influenced by socio-cultural pressures to reduce weight [9]. The same group [10] found similarly greater body dissatisfaction in urban and rural non-indigenous adolescents but this study did not report on use of weight loss behaviours. In a national study of health, eating, weight and culture among 7889 school children in every Australian state and territory, 333 were of indigenous origin. In this study indigenous females and males perceived themselves as heavier in body weight but were more likely to be trying to gain weight and build up their bodies and to be advised by parents and family to do so [11]. These mixed reports may indicate different attitudes towards body shape, and a greater desirability for muscular strength in indigenous compared to non-indigenous Australian adolescents. However, there is no information on how, if at all, these differing attitudes indicate a problem with eating disorders and weight in older adolescents and adults.

Our aims were therefore to investigate the current 3-month prevalence of eating disorder behaviours of binge eating, restrictive dieting, and extreme weight control methods such as vomiting, and core eating disorder psychopathology of excessive weight and shape concerns, in a representative general population sample of older adolescent and adult indigenous Australians.

## Methods

Two independent cross-sectional single stage interview based surveys were conducted in 2005 and in 2008. Both surveys were embedded in the respective year's Health Omnibus Survey, under the auspices of the South Australian Health Commission. The interviews were conducted by Harrison Health Research [12].

### Sample selection and interview procedures

The sample selection and interview procedures were the same for each survey. Samples were selected from both metropolitan and rural areas. For the metropolitan sample in 2005 386, and in 2008 375 "collectors' districts" were selected from those used by the Australian Bureau of Statistics in the 2001 and 2006 census respectively [13]. For the country samples, all towns of 10,000 or more in population size and a selection of towns of at least 1,000 people were surveyed. The collectors' districts were chosen according to their probability of selection proportional to size. Within each collector's district a starting point was randomly selected. From this starting point, using a pre-determined process based on a "skip" pattern of every fourth household, 10 dwellings were chosen. Only one interview was conducted per household or dwelling, and, where more than one resident was aged over 15 years, the respondent was the person whose birthday was last. The sample was a non-replacement sample, and up to six separate visits were made to interview the person chosen to take part.

The interview was piloted during August 2005 and August 2008, with 50 interviews. No formal reliability study was done, but 10% of each interviewer's work was selected at random, and the respondents re-contacted and a number of questions were asked of them, to ensure they had been interviewed as reported. Interviews were conducted from September through to 31^st ^December 2005 and 12^th ^February through 14^th ^July in 2008 respectively.

### The interview

The structured respondent-based interview comprised a range of health-related and demographic questions, including present height and weight. The eating disorder behavior and attitude questions were written by the first author (PH), and were modeled on related questions used in the investigator-based interview, the Eating Disorder Examination (EDE; [14]). The questions were embedded towards the end of the interview. Three eating disorder behaviors were assessed, namely binge eating, purging and strict dieting or fasting. Current regular use of these behaviors was defined as the behavior occurring at least weekly over the three months prior to the interview. In order to assess eating disorder attitudes respondents were asked about the importance of weight and/or shape to self-evaluation. A further question to investigate burden was asked namely assessing 'days out of role' [15]. Body mass index (BMI; kg/m^2^) was calculated from self-reported weight and height.

The specific questions relating to eating disorders in the survey were: (i) *I would now like to ask you about episodes of overeating that you may have had recently. By overeating, or binge eating, I mean eating an unusually large amount of food in one go and at the time feeling that your eating was out of control, [that is you could not prevent yourself from overeating, or that you could not stop eating once you had started]. Over the past three months how often have you overeaten in the way I have described? *Responses were made from a 4-point list of

'not at all', 'less than weekly', 'once a week' and 'two or more times a week'; (ii) *The next questions are about various weight-control methods some people use. Over the past three months have you regularly used, that is at least ONCE A WEEK, used any of the following: laxatives, diuretics (water tablets), made yourself sick, in order to control your shape or weight? *Responses were either 'yes' or 'no'; (iii) *Over the past three months have you regularly e.g. at least once weekly, or recurrently during the three months, done any of the following: gone on a very strict diet, or eaten hardly anything at all for a time, in order to control your shape or weight? *Responses were either 'yes' or 'no'; (iv) *In the past three months has your weight and/or your shape influenced how you think about (judge) yourself as a person? E.g. has it been a really important issue to you/to your self-confidence? *Responses were on a 6-point scale from 'not at all' to 'extremely (the most important thing for you)' with a cut-off of ≥ 4 to indicate at least moderate importance; and in 2005 (v) *During the past four weeks, on how many days (approximately), if any, were you unable to complete your work, study or household responsibilities because of any problem with your physical or emotional health? *The number of days between 0 and 28 was recorded. In 2008 participants were also asked about subjective binge eating and health related role impairment i.e. ... *have you felt your eating was out of control when others might not agree the amount of food was large (e.g. 2-3 pieces of bread) *and *have you been unemployed or out of work for health reasons?*

### Statistics

Data were weighted by the inverse of the individual's probability of selection, then re-weighted to benchmarks derived from the Estimated Resident Populations at 30th June 1994 (and for the 2008 survey in 2006) by age, sex and Local Government Area, from the Australian Bureau of Statistics (Catalogue No 3204.4). The stratified cluster sampling approach was taken into account during the entire statistical analysis.

Numerical data were presented as mean values and standard deviations (SD). T-tests, Mann-Whitney U and chi-squared tests as appropriate were used to test for between group differences. Multivariable logistic regression analyses were conducted to determine the likelihood of a significant difference in features between indigenous and non-indigenous eating disorder behavior controlling for values of identified secular differences in each year of study. A significance level of 0.05 was employed for all tests. Analyses were conducted using the SPSS for Windows version 18.

### Ethics

All subjects in the study gave verbal informed consent to their participation and the study was approved as ethical by the Government of South Australia Department of Health. All participants received written information about the survey prior to consent being obtained. Written consent was deemed impractical in this large low risk survey by the Department. Verbal consent was obtained by the interviewers and audited by the Department, and the oral informed consent process was approved by the research ethics committee of the Department.

## Results

### The samples

Demographic and other details of the two samples and respondents are shown in Table 1. In 2005 there were a higher proportion of women in the indigenous sample compared to the non-indigenous but gender proportions were similar in 2008. In both surveys age was significantly lower in the indigenous participants. Differences in BMI, pursuit of higher education and income favored non-indigenous participants but did not consistently differ. Also shown on Table 1 are median levels of health related role impairment which were higher in indigenous participants in 2005. The proportion of those with health related unemployment did not differ between groups in 2008.

**Table 1:** Comparative demographic and eating disorder features for the 2005 and 2008 surveys

		2005					2008	
	
	Non-Indigenous	Indigenous			Non-Indigenous	Indigenous		
						
	n = 2962	n = 85			n = 2964	n = 70		
						
	n (%)		**χ**^**2**^, **df = 1**	p	n (%)		χ^2^, df = 1	p
Female	1498 (51)	55 (65)	6.60	0.01	1519 (54)	36 (51)	0.001	0.80
Regular binge eating^1,^	^2^204 (6.9%)	14 (17%)	11.4	0.001^f3^	143 (4.8%)	6 (8.5%)	2.06	0.16^f3^
Regular subjective binge eating^1,2^	-	-	-	-	53 (1.9%)	4 (5.7%)	5.56	0.04^f3^
Regular^1 ^dieting^2^	132 (4.5%)	8 (9.4%)	4.6	0.06	97 (3.3%)	4 (5.7%)	1.33	0.29^f3^
Regular^1 ^purging^2^	45 (1.5%)	2 (2.4%)	n.a.		29 (1.0%)	1 (1.4%)	n.a	n.a
Unemployed or out of work for	-	-	-		126 (4.3%)	4 (5.7%)	0.36	0.35
health reasons								
	Mean (SD)		df, t	p	Mean (SD)		df, t	p

Age/years	45.3 (18.8)	39.2 (16.7)	3045, 2.9	0.003	46.0 (8.9)	37.4 (18.3)	3032, 3.8	< 0.001
BMI/kg/m^2 ^(n = 2813)	26.1 (5.3)	26.6 (5.6)	2811, -.99	0.32	26.5 (5.3)	28.1 (5.6)	2754, -2.1	0.04
	Median (IQ range)		Z	p	Median (IQ range)		Z	p

Annual income/x$10 K	> 5-6 (> 3-4, > 8)	> 4-5 (> 2-3, > 8)	-2.9	0.003	> 6-8 (> 3-4, > 10)	> 8-10 (> 3-4, > 10)	-0.45	0.65
Highest Education^4^	5 (3, 7)	4 (3, 7)	-0.22	0.826	5 (3, 7)	5 (2, 6)	-2.01	0.04
Weight/shape importance	0 (0, 3)	1 (0, 4)	-3.5	0.001	3 (0, 4)	2 (0, 4)	-1.03	0.30
Days out of role	0 (0, 0)	0 (0, 2)	-2.2	0.032	-	-	-	-

All results were adjusted for cluster sampling and weighted according to census data.

1. Current and regular was defined as the behavior occurring at least weekly over the three months prior to the interview;

2. Binge eating was described as episodes of overeating, namely eating an unusually large amount of food in one go and at the time feeling that the eating was out of control, (i.e. it could not be prevented or stopped) [16]; Subjective binge eating was when others might not agree the amount of food was unusually large [14]; Purging was described as a weight control method comprising the use of laxatives, diuretics (water tablets), or self-induced vomiting; Strict dieting was described as "going on a very strict diet", and fasting as "eating hardly anything at all for a time", both for the purpose of weight or shape control;

3. f = Fishers exact statistic

4. Highest education: 1 = still at school, 2 = Left school at 15 years or less, 3 = Left school after age 15 but still studying, 4 = Trade qualification/apprenticeship, 5 = Certificate/Diploma - one year full time or less, 6 = Certificate/Diploma - more than one year full time, 7 = Bachelor degree or higher.

### Prevalence of eating disorder features

As shown on Table 1, in 2005 the frequencies of current restrictive dieting and other weight control behaviors were similar in both groups. Current weekly objective binge eating was more frequent and median levels of the importance of weight and/or shape to self-evaluation were higher in indigenous participants. These differences remained significant when controlling for 2005 demographic differences (age, income and gender) on logistic regression analysis. (Regular objective binge eating OR = 2.5 95% CI 1.4-4.4 p = 0.002 and moderate or higher level of weight/shape concerns OR = 2.1 95% CI 1.3-3.3 p = 0.002).

In 2008 eating disorder behavior frequencies and levels of weight and shape concerns were similar in indigenous and non-indigenous participants. Subjective binge eating frequency was higher in indigenous participants but this lost significance when controlling for 2008 demographic and body weight differences (age and body mass index) on logistic regression analysis (OR = 2.0 95% CI 0.6-6.7 p = 0.28).

### Eating disorder features and binge eating across gender and BMI in indigenous participants

For this section statistical tests were conducted only on data with at least n = 10 per group.

Within the indigenous participants in 2005 there were 2 males and 12 females with regular objective binge eating, no males with regular purging and no males with regular strict dieting or fasting. The median level of weight and shape concern in males was 1 (IQ range 0, 4) which not significantly different to that in females (median 1, IQ range 0, 4; Mann Whitney U Z = -0.3, p = 0.80). In 2005 the mean BMI of those with and without regular objective binge eating was similar (28.3 SD 8.4 and 26.3 SD 5.0 respectively, t = -1.1, p = 0.28).

Within the indigenous participants in 2008 there were 6 males and no females with regular objective binge eating, 3 males and one female with subjective binge eating, no males with regular purging and two males and two females with regular strict dieting or fasting. The median level of weight and shape concern in males was 2 (IQ range 0, 3) which was significantly lower than in females (median 3, IQ range 2, 4; Mann Whitney U Z = -2.6, p = 0.009).

In 2008 the mean BMI of those with and without regular subjective binge eating was similar (27.7 SD 2.9 and 28.1 SD 5.7 respectively). The mean BMI of those with regular objective binge eating was greater than that of those without (31.1 SD 5.5 and 27.6 SD 5.4 respectively).

## Discussion

The present study to our knowledge is the first to provide data on eating disorder features in Australian indigenous peoples derived from a general population sample of older adolescents and adults. The findings support eating disorder problems of subjective and objective binge eating being at least as common if not more common (controlling for secular differences) in indigenous peoples. (Subjective binge eating is where the person perceives an episode of overeating on an amount of food that is not excessively large given the social and cultural context of eating. Objective binge eating is where the person perceives an episode of overeating on an amount of food that is excessively large given the social and cultural context of eating [14,16].) In addition, the core cognitive feature of an eating disorder, namely weight and/or shape concern, was also as high if not higher in indigenous participants (controlling for secular differences) and levels of restrictive dieting and other compensatory weight control behaviors were similar to non-indigenous. The small numbers of people with Aboriginal or Torres Strait islander who identified their ethnicity precluded statistical comparison of rates of purging behaviors and estimate of diagnostic eating disorder prevalence. However, it can be surmised that eating disorders are likely to be at least as much a problem for indigenous as for non-indigenous Australians. Furthermore, the similar (or greater) prevalence of binge eating behaviors were not explained by secular differences in indigenous such as younger age, higher body weight, gender or income levels.

Caution must be exercised in discussing the results of eating disorder features and gender in the indigenous participants as numbers were very small and there were differences across the two years of survey. The finding that regular binge eating (objective and subjective) was as if not more common in males in 2008 is however in accord with international studies. These have found that unlike anorexia nervosa and bulimia nervosa, binge eating is as common in males as it is in females [17]. In addition in both years we found BMI to be higher in those with regular objective binge eating. This is also consistent with research indicating a higher frequency of disordered and binge eating in the overweight and obese [17,18].

The present study was limited in that assessment was only at two time points. In addition, the survey instrument has not been validated for cross-cultural use. It also did not record details of the most common types of weight control behaviors, nor details of restrictive dieting nor the types of foods consumed during a binge and it had no assessment of excessive exercise or body composition concerns (such as a favorable regard for increased muscularity as found in previous research [11]). It would have been desirable to have included assessment of lifetime as well as current prevalence rates. It is important to note that the findings can only be generalized to indigenous Australians living in metropolitan areas with probable less income and education disadvantage than for those living in rural and remote areas. Strengths of the present study were the replication of results in a second survey within 3-years, standardized and rigorous survey methods, including random selection and interview assessment, the good response rates, wide age range and inclusion of both genders. Small numbers preclude analysis of time trends in the indigenous participants but the apparent decrease in rate of eating disorder features needs further study in larger samples and over a longer time period.

The findings are important as they highlight for indigenous Australians the problem of disordered eating and body weight concerns, eating disorder features that are commonly associated with obesity. This is consistent with the increased frequency of obesity related physical health problems such as diabetes, hypertension and the metabolic syndrome which are well recognized as contributing to shortened lifespan and greater morbidity in Aboriginal and Torres Strait Islander peoples. The poor dietary choices that likely underpin these problems can been seen in the wider context of cultural dispossession whereby traditional methods of food preparation and choices of 'plain foods' have often been displaced with pre-prepared 'fast' foods of lower nutritional value. This is exacerbated by the social disadvantage and, as indeed we found, increased health related role impairment of indigenous peoples. More research is needed to investigate the cultural and social context of food and eating and subsequent health problems in indigenous Australians.

The present study points to the need for health professionals to ask about and identify binge eating and other features of disordered eating in indigenous patients presenting with these medical problems. Not only are these important co-morbidities of obesity but they may impede weight management programs and exacerbate co-morbid medical problems such as diabetes [19] if not addressed. Furthermore, there are real concerns about the role of extreme dietary restriction and other weight control methods in exacerbating bulimic behaviors and contributing to weight gain [18]. On the other hand, psychological approaches in particular cognitive behavior therapy, are effective in reducing binge eating and other bulimic behaviors [20] and professionally lead behavioral weight management programs may attenuate eating disorder behaviors [21].

## Conclusions

It is unclear whether concerns regarding weight and shape in indigenous participants were related to health literacy regarding physical health effects of obesity and/or societal stigma towards obesity and positive regard for thinness. However, the results indicate that it cannot be said there less is weight concern or disordered eating behaviours in indigenous people. Like others with eating disorders, it is probable that indigenous Australians would be receptive to and helped by inquiry about eating disorder symptoms and advice on appropriate help.

## Competing interests

The authors declare that they have no competing interests.

## Authors' contributions

PH submitted the eating disorder questions to the survey, conducted data analysis and drafted the manuscript. CC critiqued the survey questions, provided essential cultural context and helped to draft the manuscript. Both authors read and approved the final manuscript.

## Pre-publication history

The pre-publication history for this paper can be accessed here:

http://www.biomedcentral.com/1471-2458/12/233/prepub
